# Supplementary figures and images for: Clinical-grade whole-genome sequencing and 3′ transcriptome analysis of colorectal cancer patients
Source: Genome Med. 2021 Feb 25;13:33. doi: 10.1186/s13073-021-00852-8 (PMC7908713; doi:10.1186/s13073-021-00852-8)

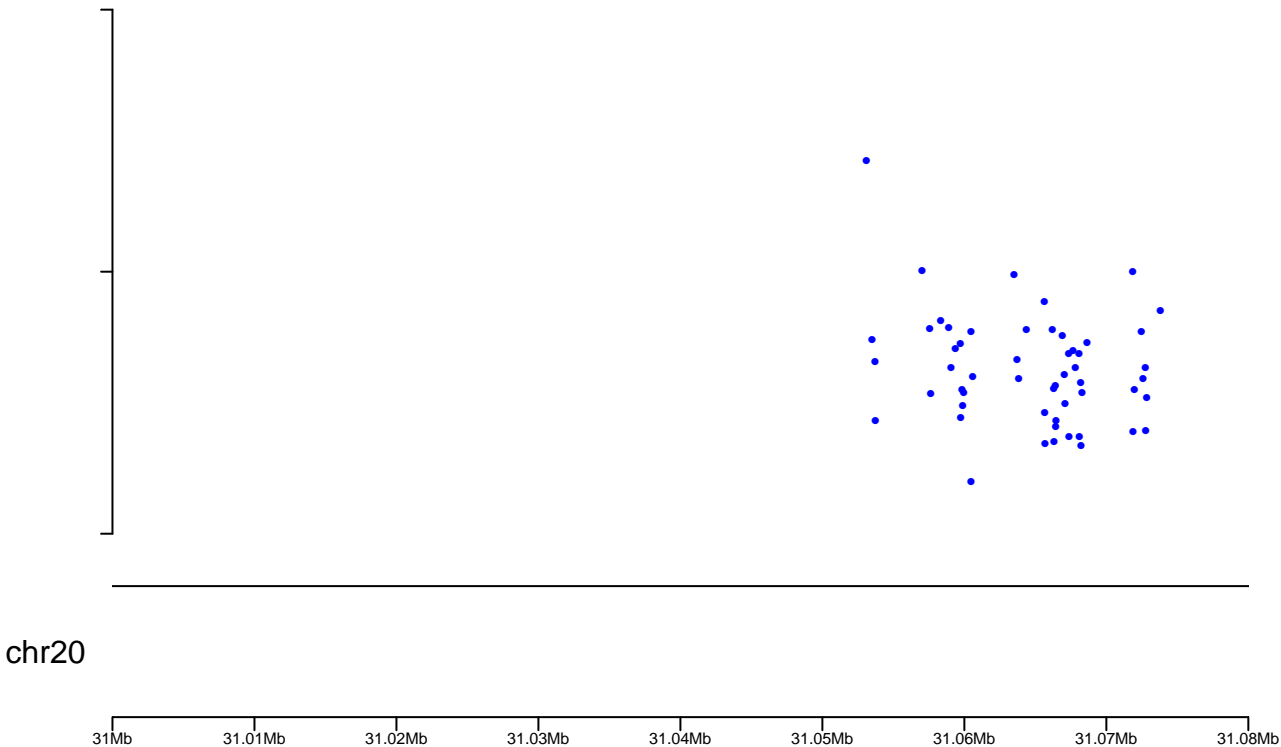

Supplement: Supplementary file 1 — Additional file 1: Figure S1. Kategis plot of Chromosome 20. Table S1. MutSigCV calls of hypermutated and non-hypermutated tumours. [file 13073_2021_852_MOESM1_ESM.zip › Supplementary figure 1.pdf]
